# Artificial intelligence‐based clinical decision support in modern medical physics: Selection, acceptance, commissioning, and quality assurance

**DOI:** 10.1002/mp.13562

**Published:** 2020-05-17

**Authors:** Geetha Mahadevaiah, Prasad RV, Inigo Bermejo, David Jaffray, Andre Dekker, Leonard Wee

**Affiliations:** ^1^ Philips Research India Bangalore 560045 India; ^2^ Department of Radiation Oncology (MAASTRO), GROW School for Oncology and Developmental Biology Maastricht University Medical Centre Maastricht 6229 ET Netherlands; ^3^ Princess Margaret Cancer Centre University Health Network Toronto ON M5G 2M9 Canada

**Keywords:** artificial intelligence, clinical decision support, machine learning

## Abstract

**Background:**

Recent advances in machine and deep learning based on an increased availability of clinical data have fueled renewed interest in computerized clinical decision support systems (CDSSs). CDSSs have shown great potential to improve healthcare, increase patient safety and reduce costs. However, the use of CDSSs is not without pitfalls, as an inadequate or faulty CDSS can potentially deteriorate the quality of healthcare and put patients at risk. In addition, the adoption of a CDSS might fail because its intended users ignore the output of the CDSS due to lack of trust, relevancy or actionability.

**Aim:**

In this article, we provide guidance based on literature for the different aspects involved in the adoption of a CDSS with a special focus on machine and deep learning based systems: selection, acceptance testing, commissioning, implementation and quality assurance.

**Results:**

A rigorous selection process will help identify the CDSS that best fits the preferences and requirements of the local site. Acceptance testing will make sure that the selected CDSS fulfills the defined specifications and satisfies the safety requirements. The commissioning process will prepare the CDSS for safe clinical use at the local site. An effective implementation phase should result in an orderly roll out of the CDSS to the well‐trained end‐users whose expectations have been managed. And finally, quality assurance will make sure that the performance of the CDSS is maintained and that any issues are promptly identified and solved.

**Conclusion:**

We conclude that a systematic approach to the adoption of a CDSS will help avoid pitfalls, improve patient safety and increase the chances of success.

## 1 Introduction

The recent prominence of artificial intelligence (AI) and machine learning (ML), coupled with the growing volume of available clinical data, has led to an increased interest in applications of AI in general^1^ and of computerized clinical decision support systems (CDSS) in particular. A computerized CDSS is any software designed to aid clinicians and patients in clinical decision‐making, defined as “active knowledge systems which use two or more items of patient data to generate case‐specific advice,” according to Wyatt and Spiegelhalter.^2^ CDSSs can make use of expert knowledge and/or models learnt using statistics and ML from data.

In the early days of CDSSs, they were conceived as being able to eventually replace the clinician's decision‐making. A nuanced, more modern view of the purpose of CDSSs is to assist the clinician to make better decisions than either the clinician or the CDSS could make on their own, by processing the vast amount of available information. Typically, a modern CDSS makes recommendations to the clinician, and the clinicians are expected make their own decisions and overruling CDSS recommendations they believe to be inappropriate. Computerized CDSS has evolved dramatically since their first steps featuring the computer‐aided diagnosis in the Leeds Abdominal Pain system,^3^ the rule‐based MYCIN,^4^ and the HELP alert system.^5^ One way they have evolved in is their integration into clinical workflows and other clinical information systems: in the beginning, they were standalone systems where clinicians had to enter the patient information before reading and interpreting the results. Beginning in 1967, CDSSs started to be integrated into clinical information management systems thus offering two main advantages: users did not have to reenter information, and CDSSs could be proactive, that is, alerting or recommending actions, without the user actively seeking assistance from the CDSS.^6^ Starting in the late 1980s, the development and adoption of standards to represent, store, and share clinical knowledge allowed separation of knowledge content from the software code of the CDSS.^7^ From 2005, clinical information systems started offering application programming interfaces (APIs) through which they could interact with CDSS, thus allowing for a more dynamic and less standardized relationship.^8^

The evolution of CDSSs has led to a high variety of types of CDSS,^9^ which can be classified in terms of a number of features. CDSS can offer support on demand or unprompted, as is the case of alert systems.^10^ In addition, CDSSs can be classified in terms of their underlying technology as based on rules, deep learning,^11^ probabilistic models, genetic algorithms, or reinforcement learning,^12^ among others. In terms of their function, CDSSs can be classified as supporting diagnosis, outcome prediction,^13^ treatment planning,^14^ prescribing and managing medications,^15^, ^16^ preventative care,^17^ chronic disease management,^18^ image interpretation (contouring,^19^ segmentation, and pathology detection), and many others.

Systematic reviews suggest that use of CDSSs reduces unwarranted practice variation, improves quality of healthcare, reduces waste in the healthcare system, and reduces the risk of overload and burnout among clinicians.^20^, ^21^, ^22^, ^23^, ^24^ However, CDSSs can also have important negative consequences, since a faulty CDSS or its inappropriate use can lead to deterioration of the quality of care. Major ethical questions and patient safety concerns still remain.^25^ The role of CDSSs has traditionally been to “enhance and support” users (clinicians or patients) who are ultimately liable for the clinical decisions.^22^ With the advent of deep learning, CDSSs are reaching human performance levels at a variety of tasks, especially image analysis, often acting as “black boxes” where the reasoning for the recommendation is unknown.^26^ This raises new questions regarding responsibility and liabilities. Regulatory processes are adapting accordingly, classifying some CDSSs as medical devices (with its legal implications) while excluding from this definition other CDSSs, such as those that do not analyze images and that allow the users to review the basis of the recommendations.^27^ However, not even regulatory approval is a guarantee of positive impact. CDSS can inadvertently increase the workload of the clinicians. For example, a well known consequence of a CDSS alerting system in patient monitoring is “alert fatigue,” that occurs when clinicians come to ignore alerts due to an overwhelming frequency of false alarms.^28^ Another potential risk arising from the adoption of CDSSs is clinicians losing the ability to make decisions on their own or to determine when it is appropriate to override the CDSS — and again current gains in artificial intelligence, which make it a reality that CDSS is equal or better in decision‐making than humans, make these risks more pertinent. This could become critical in case of computer system downtime, or if a patient with an unusually rare medical condition is admitted for treatment. As such, it is important to remain alert to both the positive and negative potential impact of CDSS on clinical decision‐making.^22^ Some forms of CDSSs have been in use for decades, but their use is not yet widespread due to a number of issues related to design and implementation, such as clinicians not using them due to lack of time or lack of confidence in the CDSS's output.^29^, ^30^

However, there remains an immense potential need for CDSSs due to increasing volume of available data, growing diversity of treatment options, and rapidly evolving medical technologies. CDSSs could be valuable as a means of delivering medical care tailored toward patients' preferences and biological characteristics. Patients could benefit from an overall accumulation of human knowledge and clinical expertise guiding their diagnosis, treatment, and condition monitoring. There remains a growing global need for high‐quality personalized medicine to improve patient outcomes, reduce financial burden, and avoid unwarranted practice deviations. Machine learning‐based CDSSs are expected to help alleviate some of the current knowledge and associated quality of care variation across countries and regions. Thus, the question of designing, developing, presenting, implementing, evaluating, and maintaining all types of clinical decision support capabilities for clinicians, patients, and consumers remains a key area of research in modern medicine.^31^

The aim of this paper is to provide guidance on the different stages for a safe and successful adoption of CDSSs (see Table 1) in a clinic safely and successfully. The paper is organized as follows: the next section explains how to select a CDSS; the next two sections provide recommendations for the acceptance testing and commissioning of a CDSS; then, the implementation section describes how to roll out a CDSS while Section 66 provides guidelines for the quality assurance of CDSSs; finally, we draw some conclusions.

**Table 1:** Summary of stages in the adoption of a CDSS.

Stages	Objective
Selection	Pick most appropriate CDSS in terms of match with target use case and clinical workflow, five “rights,” performance, and user acceptability
Acceptance testing	Test that CDSS satisfies security, privacy, and safety requirements applicable to medical devices, covering typical error scenarios, exceptions, and unforeseen conditions
Commissioning	Prepare the CDSS for optimized use in the clinic (including potential customization) and test its safety and performance within the local context
Implementation	Roll out the CDSS and transition from the old workflow to the new after training the end users and managing their expectations
Quality assurance	Ensure that the quality of the CDSS remains fit for purpose by monitoring internal and external updates as well as context drift

## 2 Selection

The range of commercially available CDSSs for clinical applications has been growing during recent years. Hence, selecting the most appropriate CDSS from those available is not always easy, yet it is a key step in the implementation of a successful CDSS.

User acceptance of CDSS is critical; several implementation studies^32^, ^33^ show that how beneficial a CDSS is perceived largely determines uptake and usage by clinicians and allied health professionals. Therefore, the recommended first step in the process would be to form a multidisciplinary steering committee comprising key clinical stakeholders, such as a number of clinician “champions,” patient representatives, department administrators, and information technology experts, who would be willing to take decisions and be accountable for the implementation of a CDSS.^34^ Studies show that likelihood of user acceptance increases when CDSS implementation involves the end users instead of forcing the CDSS onto the end users.^32^ In order for the CDSS to be effective, the CDSS should be conceived as part of a wider, coherent, and department‐wide quality improvement strategy, where a clinical quality gap between current patient outcomes or process and the desired end state has been clearly identified and carefully measured.^35^

Two main aspects to consider when selecting a CDSS are the quality of the CDSS and how well the CDSS fits with closing the clinical quality gap. The quality of a CDSS needs to be considered at least at two levels: the level of the technology platform and that of the data or knowledge used to build it. CDSSs, as software that is potentially also a medical device, should be designed, implemented, tested, and documented using generally recognized quality assurance methods for software development used in the medical domain. The medical knowledge used in the construction of the CDSS cannot be proven clinically complete or objectively correct, but it must attempt to capture the current state of professional and scientific opinion. Furthermore, it must be possible to verify formally that the relevant medical knowledge satisfies certain requirements such as being unbiased, consistently interpreted, and reasonably completed.^36^ In the case of CDSS based on models learnt using statistical analysis or by machine learning, an assessment of the quality of the source data is necessary. Data quality is important, since the “garbage in, garbage out” principle especially applies to machine learning. Data are generally defined as of high quality if it fits closely to the intended purpose,^37^ and more specifically it should consist of a representative, unbiased sample of the domain (patients or clinical conditions) being modeled. The appropriate processes for anomaly detection, data cleansing, and handling of incomplete or missing data should have been applied to the dataset, and the existence of potential biases assessed and corrected.

A key indicator of the quality of a CDSS is its performance. Measures of performance vary across different types of CDSS. For example, in CDSS performing outcome prediction, the area under the receiver operating characteristic (ROC) curve or the c‐index is commonly used performance metrics.^13^ In other cases, performance can be measured in terms of saved time.^19^ However, the assessment of the performance might be complicated,^38^ especially when a gold standard of performance does not exist, such as in the case of therapy‐advice systems, where even experts may disagree. In the end, the most difficult to measure, yet most valuable performance metric, is the effect of the CDSS on health outcomes or processes.^35^ Publication by CDSS vendors of detailed evaluations of usability and effectiveness of CDSS implementation might facilitate purchasing decisions,^34^ but it should be kept in mind that trials conducted by developers of CDSS might overestimate their benefits, and third party external validation is required.^39^ A thorough hazard analysis, resulting in an exhaustive list of potential risks and their possible consequences along with a mitigation plan for said risks,^36^ is part of the regulatory process and could provide valuable insights into the desirability of the CDSSs.

During selection, the acceptability of the CDSS should be considered and weighed against performance. For users to easily accept the output of a CDSS, the strength of evidence supporting the clinical recommendations delivered by the CDSS should be transparent to the user^40^. The levels of comprehensibility or explainability of models based on hand‐engineered features and simple models (e.g., decision trees) are usually higher than those based on more advanced approaches such as random forests and deep learning.^41^

As mentioned earlier, it is crucial to select a CDSS that fits the requirements of the local site. First, following the Population, Intervention, Comparison, and Outcome (PICO) framework,^42^ the selection process should be restricted to CDSSs that target the appropriate population, consider the relevant intervention and comparators, and focus on the outcomes of interest. When selecting a CDSS, we should consider the five “rights” a CDSS should fulfill, namely: delivering the right information (what), to the right people (who), in the right format (how) through the right channels (where) at the right time in the workflow (when).^43^ Delivering the right information also implies that the output of the CDSS (clinical recommendations and assessments) should be clinically relevant, brief, unambiguous, and actionable.^40^ The CDSS should also fit the existing workflow of its users as closely as possible, for example, integrated in the electronic health record (EHR), minimizing the effort required by users to receive and act on system recommendations.^44^. In order for a CDSS to fit the workflow of a particular clinic, customization of the CDSS might be necessary. Therefore, the customization functionality offered by each CDSS should be taken into account during selection.^28^ Another consideration related to the local workflow is whether all the necessary data for the proper functioning of the CDSS is available in that specific point in the workflow.^45^

Another factor to consider when selecting a CDSS is its usability, more specifically how easy is it to use or how much training is needed to be able to use the CDSS. Vendors need to be clear about the expertise required for using the system.

An important consideration when selecting a CDSS should be its cost‐effectiveness,^46^ compared to alternative CDSS or even other medical devices (e.g., a new piece of equipment). However, it remains difficult to demonstrate the return on investment of CDSS, especially against many competing priorities at the delivery system level.^34^ A comprehensive assessment of the costs involved in the acquisition of a CDSS should be undertaken prior to its purchase, including one‐off costs (purchase, training, implementation, etc.) but also costs incurred over time such as maintenance costs and resource utilization (e.g., time of its users). These costs should be weighed against not only estimated improvements in health outcomes but also estimated savings due to efficiencies facilitated by the CDSS.

Other factors to consider include the compatibility with legacy applications, the maturity of the CDSS, and the availability of upgrades.^23^

## 3 Acceptance testing

For acceptance testing, a CDSS can best be seen a medical device for which many processes are already usually in place in health‐care providers. Acceptance tests for a medical device assure that the all defined specifications are fulfilled and that the medical device satisfies pertinent safety requirements.^47^ These tests are usually defined by the CDSS vendor, but should be run in the presence of the representatives of the local site. On successful completion of the acceptance tests, the acceptance report will be signed and the payment for the device approved. Consequently, the set of test cases should be comprehensive, including covering cases on the edge of the domain of the CDSS, usually termed corner cases. The technical aspects of acceptance tests should be conducted by technology representatives while tests focused on usability or clinically oriented tests should be conducted by a subgroup of users that comprises a representative sample of the intended end‐user population. The acceptance test plan should cover at least the following aspects:

1. Installation and setup of the device.
2. Proper functioning of APIs offered by the CDSS (if any).
3. A complete walkthrough of the user interface, operating the CDSS as part of the existing workflow.
4. Clinical completeness, relevance, comprehensibility, consistency, and repeatability of the output of the CDSS.
5. Auditing, security, and privacy functions.
6. Typical error scenarios, such as unexpected, incorrect or incomplete input data, abrupt closure scenarios (e.g., due to power outage) leading to incomplete transactions, etc. The CDSS should not output inappropriate recommendations in the event of incomplete or inaccurate data. Moreover, the CDSS is expected to handle these situations by keeping internal consistency, providing appropriate error messages, and, if necessary, proceeding to an orderly shutdown.

In addition to the above, acceptance testing of a CDSS should test the accuracy of the CDSS recommendations, as inaccurate recommendations might endanger the safety or well‐being of patients. These tests should compare the outcome of the CDSS to the expected outcome on a fixed, small, and restricted but representative sample of real cases. The estimated accuracy based on these acceptance test results should be compared against the accuracy claimed by the vendor and statistically test whether it is within the specified error tolerance. The same applies to the other quantitative and qualitative estimates provided by the vendor. In order to test whether the real accuracy of the CDSS (or any other parameter) is within a given error tolerance based on a sample of tests, a statistical test (e.g., Mann–Whitney *U* test) should be used to calculate the probability that the accuracy observed in the sample belongs to a probability distribution determined by the claimed accuracy and error tolerance. If the calculated probability is below a certain significance threshold, we can reject the hypothesis that the actual accuracy is within the error tolerance. Finally, a check for completeness and accessibility of the CDSS user manual as part of acceptance testing would be important for novice users or in emergency, unusual situations.

## 4 Commissioning

Commissioning is the process that prepares the CDSS for safe clinical use in the local site, meeting established requirements and end users' expectations.^48^ As such, commissioning verifies that the CDSS has been installed in the local site following the agreed requirements, successfully handed over from the vendor, and most importantly, that it functions properly. It is widely recommended to prepare for this phase by devising a commissioning plan that describes the tasks, schedule, and required human and equipment resources as well as the amount of support required from the CDSS vendor.

The first step in the commissioning plan is the installation in the local site, which in the case of CDSSs inevitably requires some degree of configuration or customization. Customization might be required for technical or safety reasons, for example, to make sure that parameters in the CDSS are correctly linked to the local EHR and that the definitions of clinical terms are in sync between the CDSS and local EHR. Customization is also a powerful tool to make the output of the CDSS more relevant, useful, and safe for use.^39^ A qualitative study found that all successful sites devoted considerable staff time to customization of their CDSS.^45^ An example of customization could be to assess and improve the appropriateness of alerts to avoid alert fatigue.^10^

In order to test that the installed CDSS functions properly in the local site, a test plan needs to be designed and executed. To begin with, the implementation of the CDSS is likely to require some changes in the workflow on the users end. In that case, the information necessary to support the future workflow needs to be identified and the new workflow tested. Once the new workflow is established, the aim is to ensure that the CDSS is functioning properly by testing as many clinically relevant scenarios and corner cases as possible. The steering committee formed by clinicians, administrators, and information technology experts should be involved in identifying all the relevant situations and corner cases where the installed CDSS could fail in the local site environment and lead to poor quality or reliability. A set of past cases, which includes difficult and rare cases along with a representative sample of the local case population could be retrospectively tested if a database with past cases exists. In this case, the recommendations of the CDSS is either assessed by a panel of clinical experts in a blind study where the experts ignore the CDSS's output or compared against the decisions that were taken in the past. However, is important that CDSS should be tested on real‐world cases from the users' own clinical practice prior to implementation.^45^ An option is to test the CDSS prospectively by running a pilot program where the CDSS is used in parallel to the existing workflow or where the CDSS is used with supervision using the existing workflow as fallback.^49^ Strategies to cover a representative sample of usual and rare cases include random sampling, input selection, and control flow testing.^50^ During the pilot, it is interesting to perform an initial assessment of the clinical relevance of the CDSS in terms of user acceptance, adherence to the CDSS's recommendations, and its impact on the clinical decisions and ultimately on patient or health outcomes. Significant deviations on the estimated performance of the CDSS during this phases as compared with that in acceptance testing or vendor's claims of performance and error tolerance should be discussed with the vendor. Failure mode analysis is an important part of commissioning testing, where faults in data entry are simulated and the behavior or CDSS is analyzed and tested for consistency.^51^ Testing during commissioning is also important to grow confidence of local physicians in that the support system works in their local setting.^13^

## 5 Implementation

The implementation process is an important factor in the success of a CDSS^52^ and consists of the design and execution of the rollout plan, transitioning from the old workflow to the new one including the CDSS and the deployment of the CDSS within the local site. An effective implementation of CDSS requires preparing both users and the local site's infrastructure for the widespread use of the CDSS. The preparation of the infrastructure will vary across CDSSs and local sites, but there are common themes on how to prepare users for the use of a new CDSS. Prior to and surrounding implementation, it is important to communicate with and educate the affected users.^53^ Effective training of all the stakeholders and intended users of the CDSS is key to its success 54 and should comprise different aspects such as when (and when not) to use it, how to use it, how to interpret the output of the CDSS, and when to override the CDSS recommendations, among others. It also includes helping users understand how the CDSS will impact their daily activities and how they can provide feedback.^53^ It is important as part of the training to manage users' expectations in terms of efficiency and effectiveness and make sure users understand the strengths and limitations of the CDSS.^22^ Different stakeholders might have different expectations: some primarily view CDSSs as a vehicle for promoting standardization, quality, and safety while clinicians might see it differently.^45^ Training should also serve the purpose of preparing users for a necessary leap of faith: a CDSS will only be used if it is perceived as beneficial by those using it, but the benefits of the CDSS will be appreciated only after overcoming the initial challenges of using it.^33^ Hands‐on training is a valuable tool, as users might need some handholding at first, as is on‐site support from vendors as needed to help with any immediate issues that may occur.^53^ The deployment or rollout of the CDSS can be undertaken incrementally (e.g., rolling it out in a single post or facility to “get the kinks worked out”) or all at once, which requires good preparation.^32^

## 6 Quality assurance

Before the CDSS has been deployed, it is crucial to design a quality assurance (QA) program to ensure that the performance and safety of the CDSS are maintained by assuring that its quality remains fit for the purpose throughout its life cycle.

As part of the QA program for a CDSS, performance must be defined using a set of metrics in terms of efficiency and efficacy so that the impact of the CDSS can be measured over time.^45^ Measures of efficacy might be specific to the functioning of the CDSS (e.g., sensitivity and specificity for a diagnostic tool) or generic, such as patient safety and change in health outcomes (such as life expectancy). Efficiency can be measured in resources saved, such as costs and productivity.^34^ In order to assess the CDSS performance, it is especially valuable to quantify baseline performance levels (i.e., before the implementation of the CDSS) as well as have an estimate of the target performance upfront.^53^

The QA plan must guarantee that any malfunctions are identified and resolved in the shortest time possible. To facilitate the discovery of CDSS malfunctions, mechanisms need to be in place for receiving user feedback and acting on it.^55^ Besides, CDSS malfunctions can be identified by a combination of qualitative and quantitative analyses (e.g., of firing rates for alert systems or overrides for recommender CDSS).^28^ Visual detection and statistical process control analysis have shown good results as tools to detect malfunction.^56^ In addition to malfunctions, it is important to log or track the cases where the CDSS was not adhered to (such as when an alert was ignored or a recommendation overridden), as knowing how often the CDSS is being overridden and why can offer valuable insights and lead to an identification of previously undetected malfunction.^45^ Similarly, monitoring proper utilization of the installed CDSS is important as this could lead to a reduced performance.^22^

At the data quality front, local sites have to define and enforce internal standards to assure the integrity of entered data.^45^ Data providers to the CDSS should be trained about the importance of high‐quality data and their responsibility in assuring its accuracy.

The QA plan must also assure that the performance and safety of the CDSS are maintained over time. In this sense, a CDSS is not radically different from a treatment planning system or a radiotherapy linear accelerator, because deviations of CDSS performance beyond certain bounds of tolerance have the potential to cause medical mistreatment. For example, Nakatsugawa et al.^57^ observed the need to update the prediction models with prospective data collection for maintaining the performance of their RT‐induced toxicity prediction models. The first concern in this aspect is ***external (context) drift*** over time. The patterns of clinical practice are constantly evolving over time: changes in the clinic are sometimes radical (such as the introduction of image‐guided radiation therapy or robotic surgery) and gradual at other times (e.g., percentage of patients with oropharyngeal squamous cell carcinoma expressing the p16 protein from human papilloma viral infection). Changes in patient case mixture, obsolescence of certain drugs and treatments, and recoding of prognostic clinical features and clinical guidelines based on new randomized trials could all lead to unwanted divergence of CDSS recommendations over time. Such changes are often impossible to forecast during CDSS acceptance testing and commissioning and represent potential sources of time‐dependent inconsistencies that violate the original assumptions built into the CDSS. These shifts can be related either to the input of the CDSS (e.g., clinical presentation of patients changing significantly since the CDSS was initially commissioned, thus exposing a previously unknown systematic bias toward certain patient subgroups) or its output (whereby the CDSS makes recommendations that are not in line with the most recent clinical guidelines). One other potential source of temporal divergence is ***internal (model) drift***. The models underpinning the CDSS are likely not to remain static, but be updated at specific times well after commissioning of the original CDSS. In addition, models developed on limited sample sizes may initially incorporate some systematic bias that will be gradually reduced over time as the models are fed with progressively larger datasets on which to train and validate on. As described elsewhere,^58^ models could be updated via any one of the following: (a) shifting either the baseline risk level or (for the case of binary models) the cutoff value for binary outcome, (b) computing new values for an existing set of parameters, or (c) the model is trained afresh on expanded data, leading to possibly new model parameters, new coefficients, and (for binary outcomes) new cut‐off values.

A suitable safeguard for internal and external drift is to establish and routinely review incident monitoring logs for inappropriate or incorrect responses from the CDSS. At the same time, a “repeated local validation” cohort should be assembled from time to time or preferably continuously to critically reexamine the tests done during the commissioning stage. The repetition may help to ensure that the CDSS remains clinically valid, despite changes in local practice or evidence‐based guidelines. Such a continuous local validation infrastructure will also be beneficial when introducing an update to the CDSS (see below). Finally, it is important to reemphasize that no CDSS can ever be perfect, but at the very least, the quality assurance system will document that the performance of the CDSS meets criteria based on the commission results as a benchmark.

Among the top priorities for the CDSS, steering committee would be to establish an update management protocol. CDSS, in common with medical software in general, is most likely to be updated in the “offline” mode. That is, via a vendor‐instigated or user‐instigated change request, a CDSS is temporarily taken out of clinical use and placed in “maintenance” mode. Subsequent changes are performed in the maintenance state, such as applying a software version upgrade or correcting of faulty function. In analogy with other aspects of maintenance and QA of clinical systems, “clinical hand‐over,” that is, acceptance of the system back into clinically operational mode, following any such update should only be allowed after some CDSS performance verification checks have been performed on the changed system. The minimum necessary tests should have been prespecified by the vendor or the maintenance manual based on risk analysis, but it may be advisable to include some additional tests taken from the acceptance testing procedures, in order to certify that all of the essential functionality of the CDSS has been restored following the update. With migration of medical software systems to “cloud services,” increasing system automation and mathematical algorithms that are able to learn “on‐the‐fly,” one also has to countenance the possibility of CDSSs that update “online.” Such CDSSs can be allowed to evolve in real time based on interactions between the user and its recommendations, such that the behavior of the CDSS might slightly change with each interaction. An update management protocol may explicitly permit online updates, which pose a new and interesting challenge, that of seeking the ideal trade‐off between the potential of continuous improvement of performance against the risk of undetected performance degradation due to, for example, systematic biases in the input.

Another top priority should be to implement a routine QA test schedule that specifies which tests should be done, when they should be done and by whom.^53^ As part of the QA tests, various aspects of the functionality of the CDSS are tested against an agreed upon ground truth. As a general rule, the types of QA tasks are drawn from the same checks as for commissioning. Therefore, the documented results of commissioning can be reused at specified time intervals, in order to certify that the CDSS performance has not unduly drifted over time. Multiple statistical anomaly detection models applied to anomaly detection on CDSS over time have been described and compared in the literature, and the most appropriate method will depend on the nature of the CDSS.^59^, ^60^ The nature and frequency of such QA tests depends on the likelihood of unwanted deviation in CDSS performance and its potential consequences. QA tests should be performed more frequently for either highly likely failures or nonconformance events that lead to severe consequences. Unlikely failures and events that do not have major clinical consequences need only to be checked infrequently. An important effort should be directed toward procedural mitigation of rare failures that carry severe consequences, because this may not be easy to intercept within a routine QA program.

In order to adequately design and execute the QA plan, it is recommended that the personnel in charge are in possession or acquire for the task a set of statistic and data analysis techniques through training. Similarly, having in‐depth knowledge of how the CDSS's underlying technology works will allow medical physicists to identify malfunctions and understand their cause. This training can potentially be offered by the vendor itself or third parties offering specialized training. Eventually, artificial intelligence and machine learning aspects will be covered within the medical physics curriculum,^61^ which will lead to a wider and deeper understanding of these systems.

## 7 Discussion and conclusions

CDSSs have shown great potential for improving healthcare and patient safety as well as reducing unwarranted variation, resource use, and costs. AI‐based CDSSs have recently stood out for their ability to leverage the increasing availability of clinical data to assist clinicians and patients in a wide variety of situations (e.g., by providing personalized estimates of clinical outcomes or proposing diagnoses) based on structured (e.g., EHRs) and unstructured data (e.g., medical imaging). However, an inaccurate or inappropriate CDSS might deteriorate the quality of healthcare and put patients at risk.^25^ AI‐based CDSSs come with additional pitfalls, including (but not limited to) overfitting to and bias and limitations in the data used to train the AI. These could lead to the CDSS's failure to generalize from the training data and ultimately to undetected poor performance at the local site. Therefore, considerable care must be taken to minimize the potential adverse consequences of CDSSs.^62^ It is important to select carefully the CDSS that matches the requirements of the local site. As with any other medical device, CDSSs require stringent acceptance testing, commissioning, and quality assurance by the local site.^13^ In addition, an effective implementation plan is key to overcome barriers for a successful CDSS.^63^ In the present review, we have summarized the guidance collected from the literature in order to provide CDSS implementers. We conclude that following a systematic approach to the different aspects involved in the adoption of a CDSS will help avoid pitfalls, improve patient safety, and increase the chances of success.

## Conflicts of Interest

The authors have no conflicts to disclose.
